# A History of Egyptian Mummies, &c. &c.

**Published:** 1834-07-01

**Authors:** 


### A HISTORY OF EGYPTIAN MUMMIES, AND AN ACCOUNT OF THE WORSHIP AND EMBALMING OF THE SACRED ANIMALS BY THE EGYPTIANS, WITH REMARKS ON THE FUNERAL CEREMONIES OF DIFFERENT NATIONS, &c. &c. By *Thos. Joseph Pettigrew*, F.R.S., &c. &c. 4to. with Plates, 1834.

THE labours which have been expended on the dissection and decyphering of mummies require new terms to designate their nature. Mummiotomy may be allowed for the former—and mummiography for the latter. The various discourses and disquisitions respecting embalming, and funereal ceremonies, may come in under the head of mummiology. Mr. Pettigrew and Dr. Granville have taken great pains to make us acquainted with these sacred or superstitious operations for preserving the frail and otherwise perishable tenement of man, which occupied so much of the time and attention of the Egyptian priesthood and the aristocracy of the Nile. These investigations are certainly both curious and interesting, not only in relation to the history of the human species; but to the condition of the arts and sciences at those remote periods when embalming the dead was so much practised.

Although our more immediate concerns are with the living and the recently dead, yet we think a rapid sketch of the prominent features of the work before us will not prove unacceptable to our readers, especially when it is remembered that the (necessary) price of the volume must render it inaccessible to the great mass of medical practitioners.

We shall not dwell on the etymology of " mummy," whether the term be confined to the body preserved or to the preserving body—the inquiry ending, like too many of the same kind, in conclusions where nothing is concluded. Mr. Pettigrew employs the term as signifying the body embalmed.

In the 16th and 17th centuries mummy formed a drug of no very uncommon prescription. The mummy, including its bituminous wrappings, was considered as having the power of throwing off from the stomach collections of congealed blood. When this and many other valuable qualities of the mummy were verified—and what may not be verified as to drugs?—

" Some Jews entered upon a speculation to furnish the mummy thus brought into demand as an article of commerce, and undertook to embalm dead bodies and to sell them to the Christians. They took all the executed criminals, and bodies of all descriptions that could be obtained, filled the head and inside of the bodies with simple asphaltum, an article of very small price, made incisions into the muscular parts of the limbs, inserted into them also the asphaltum and then bound them up tightly. This being done, the bodies were exposed to the heat of the sun; they dried quickly, and resembled in appearance the truly prepared mummies. These were sold to the Christians." 8.

The mummy seems to have been a favourite remedy among our Gallic brethren, and Francis the First was in the habit of always carrying some mummy-powder about with him, ready to take upon receiving any wound or injury! Armed with this universal panacea he considered himself secure against all danger! And no wonder, when Avicenna, one of the lions of antiquity, avers that this said mummy is very useful in fractures, paralysis, hemicrania, epilepsy, hæmoptysis, palpitation of the heart, diseases of the liver, and dozens of diseases for which even veratria has not been pronounced as a specific. The GREAT Lord Bacon informs us that "mummy hath great force in stanching blood;" while the GREATER Ambrose Paré assures us that it was a remedy of high reputation in his day. We have applied the term "GREATER" to Paré, because that shrewd surgeon condemned the remedy as perfectly useless. We must not laugh too heartily at the *mummeries* of the olden time, seeing that we have abundance of them in our own days. Have we not a Bacon in Harley Street, and an Avicenna in Hatton Garden, vouching for the efficacy of more pernicious drugs than a piece of powdered mummy?

From physic let us make a short digression to metaphysic.

"Believing in the immortality of the soul, the ancient Egyptians conceived that they were retaining the soul within the body as long as the form of the body could be preserved entire, or were facilitating the reunion of it with the body, at the day of resurrection, by preserving the body from corruption." 13.

The creed of the Egyptians was this:—that when death occurs, the soul enters into some other animal just born; and, after going the round of all animals on the earth, in the air, and in the waters under the earth, occupying a period of about 3000 years, *more or less*, the soul again enters the body of a man *just born*. Now upon this creed it is needless to say that the soul of the departed mummy could never again enter its pristine habitation, if that habitation were preserved three million of years instead of three thousand! The theology of mummies, we fear, must come down to an humbler *rationale* than this absurd theory affords us—which is indeed in direct contradiction to the principles on which it is said to be founded. Even if the belief of a *resurrection* prevailed among the Egyptians, which we very much doubt, the practice of embalming, *on that account*, must have been equally absurd. The power that called up the body to be re-inhabited by its former soul, would not need the power of pitch or asphaltum to work this miracle; and if it *did* need this mummery, what was to become of the myriads who could not afford the expense of embalming?

The whole affair, in our humble opinion, resolves itself into a simple principle, which is as operative at this moment as in the days of Sesostris—the wish and the endeavour to prolong the memory of our friends and of ourselves. This "longing after immortality" is deeply mixed up with our nature, and shews itself in life, in death—nay, after death. No one can pass through a country church-yard without remarking the "frail memorials" of extinguished existence, erected by friends or even by the departed themselves, to procrastinate that oblivion that rolls its tide over all things. It is true that, in ancient—perhaps in modern times, the pride of kings and the cunning of priests have ingrafted on the above-mentioned natural and pious principle, a great many other principles and superstitions that have disgraced the intellect of man, and perhaps increased the sum of his physical miseries. Hence the stupendous pyramid rose, by the hard labour of millions, to inclose the bitumened corpse of some tyrant monarch! Hence the myriads of sacred animals, fostered during life, to impose on the credulity of the ignorant, and preserved, after death, to keep up the delusion, and enable the priests of Egypt to feed on the earnings of the poor. Although our modern priests are free from any blame, as to the disposal of the dead, we conceive that modern sepulture is not entirely free from the charge of superstition, folly, or pride. The *consecration* of the ground where our clay is to mingle with mother-earth may be an amiable, but it is certainly not a philosophical feeling. Can such a rite benefit the soul of the dead? We apprehend that none but a Catholic can think so—and not even he, if capable of reasoning rightly. But the practice of erecting magnificent mausoleums, such as Hadrian's and Metella's tombs, in former times, and costly sepulchres (which shall be nameless) of more modern date, cannot be contemplated without pity for the weakness of human nature. By these observations, however, we should be sorry to censure the laudable custom of erecting statues or other mementos to departed genius and merit. These encourage the virtues and excite emulation, without any reference to other states of existence. It is always dangerous to connect any rite or ceremony on the lifeless clay with the prospect or condition of the immortal soul. This, in fact, is embracing the worst parts of monkish superstition. These reflections have grown out of the disquisition contained in Mr. Pettigrew's work—a disquisition which is curious, and even amusing, considering the *grave* subject of it.

The fourth chapter, on "Egyptian tombs," contains a great deal of curious research. The fifth chapter is "on Embalming." It may be stated in a very few words, that this useless art is not practised by the modern Egyptians—and that the ancient art is unknown. Little that is precise or practical can be gathered from the accounts of embalming recorded by Herodotus or Diodorus Siculus—and we fear that even Dr. Granville and Mr. Pettigrew have given us conjectures rather than certainties, as to the medicaments by which these mummies were preserved. Our present author observes that—"one cannot but express regret that the present state of chemical knowledge is not sufficiently advanced to be capable of detecting the precise nature of the substances, chiefly of a vegetable kind, that have been used in this ancient operation." We wish well to the advancement of chemistry; but we confess we shall not go into mourning if even a Farraday should fail to analyze the preservative materials of a mummy. Passing over a very amusing chapter on the amulets, idols, implements, &c. found in Egyptian mummies, and also the chapter on sarcophagi, together with one on the Papyri manuscripts, we come to an interesting chapter on the "physical history of the Egyptians." The varieties of form and of complexion in the human race have engaged the attention and puzzled the brains of philosophers, poets, and physicians in all ages, and the matter is still involved in almost cimmerian darkness. Neither race nor climate, singly, will account for the astonishing differences of complexion in man. We doubt whether the Ethiopian would ever change his hue, if he and his descendants inhabited the North Pole for a hundred generations. The character of Egyptian physiognomy is distinctly preserved in the mummies. These resemble the Arabs rather than the Copts, Chinese, or Negroes. Mummies of the higher orders of society reveal the almost living lineaments of a people, tawny, not black, with long, and sometimes lank hair, with features which bear no trace of Negro descent. Neihbuhr, Browne, and others, consider the present Copts as the genuine descendants of the ancient Egyptians. Mr. Madden, however, is of a different opinion, and comes to his conclusions from a careful admeasurement of the heads of mummies and those of Copts. Mr. M.'s testimony rests on more accurate data than those of brother travellers or philosophers, and is therefore of more weight. One thing is clear—that the Egyptians, as far as we can judge by the mummies, were not descendants of the Negroes.

The 12th chapter gives us an account of the sacred animals embalmed by the Egyptians. Here it is that priestcraft and superstition—two words which might be almost considered synonimous—beam forth in all their glory!

"Certain animals were supported at the public expense. The people offered up presents to the animal representing the divinity to whom they were addressing their prayers, to render their supplications fruitful. Animals of the lowest character, even vile insects, have been fostered in their temples, nourished by their priests, embalmed after death, entombed with pomp, and received all kinds of honours. Those who either by accident or design have occasioned the death of any of these animals have even paid the forfeit of their lives as the penalty of the offence. 'He who has voluntarily killed a consecrated animal,' says Diodorus Siculus, 'is punished with death; but, if any one has even involuntarily killed a cat, or an ibis, it is impossible for him to escape capital punishment; the mob drags him to it, treating him with every cruelty, and sometimes without waiting for judgment to be passed. This treatment inspires such terror that if any person happens to find one of these animals dead he goes to a distance from it, and by his cries and groans indicates that he has found the animal dead." 170.

The bull, the dog, the hawk, and the ibis, were great divinities in those days. But, in some cities, the sheep, the goat, the sparrow-hawk, the crocodile—almost every creature that flew, ran, creeped, or swam, came in for divine honours, and received the expensive process of embalming!

Truly if this veneration for animals and mania for embalming human bodies had continued, Egypt would, by this time, have become one general sepulchre for dried mummies of men and beasts! The following is the conclusion to which the learned Dr. Pritchard has come respecting the Egyptian worship of various animals.

"We have seen that all the operations of nature were ascribed by the Egyptians to certain demons or spiritual beings, who were supposed to animate different portions of the universe. All these were emanations from the universal deity or soul of the world. This doctrine was extended still further; and it was imagined that the soul or vital principle in every living being is an emanation from the same source, that it is a divided portion of the divine nature, and derived either primarily or secondarily from the fountain of divinity. Accordingly, in men and animals, and even in plants, they adored the indwelling portions of the same essence." 174.

But our readers will be ready to say—satis superque, in respect to Egyptian mythology. The subjects are all illustrated by appropriate plates, and we think that Mr. Pettigrew has completely exhausted the inquiry as to mummies.

Our talented and indefatigable author, indeed, must have expended immense pains upon this dry, though curious subject of investigation—more, we freely confess, than we should have done. The work, however, will transmit his name to posterity, and will prove a valuable acquisition to the cabinets of the curious throughout the whole empire, and even far beyond the limits of the British Isles.

